# Supplementary material for: Proteotranscriptomic Profiling of the Toxic Mucus of Kulikovia alborostrata (Pilidiophora, Nemertea)
Source: Toxins (Basel). 2024 Dec 26;17(1):5. doi: 10.3390/toxins17010005 (PMC11768458; doi:10.3390/toxins17010005)
Supplement: Supplementary file 1 [file toxins-17-00005-s001.zip › Supplementary File S4.zip!/ORF 001200 LINALB/1362d454f90accc2/dom_report.pdf]

Email	vas9i-kz@mail.ru
Description	ORF_001200_LINALB
Date	Fri Apr 7 03:17:43 BST 2023
Unique Job ID	1362d454f90accc2

Domain analysis

Rank	Aligned region
1	c2f2jA_
2	d1n1ua_
3	c1n1uA_
4	c7k7xA_
5	c4ttoA_
6	c4ttmA_
7	d1nb1a_
8	c1nb1A_
9	c2khbA_
10	c7lhcA_
11	c2mn1A_
12	c4ttnA_
13	d1pt4a_
14	c2kukA_
15	c7rfaA_
16	c1jjzA_
17	c1k48A_
18	c2f2iA_
19	c2jwmA_
20	c3e4hA_
21	
